# Endothelial nitric oxide synthase, vascular integrity and human exceptional longevity

**DOI:** 10.1186/1742-4933-9-26

**Published:** 2012-11-15

**Authors:** Annibale Alessandro Puca, Albino Carrizzo, Anna Ferrario, Francesco Villa, Carmine Vecchione

**Affiliations:** 1 Cardiovascular Research Unit, IRCCS Multimedica, Milan, Italy; 2 Department of Medicine and Surgery, University of Salerno, Salerno, Italy; 3 Vascular Physiopathology Unit, IRCCS INM Neuromed, Pozzilli, (IS), Italy; 4 Instituto di Tecnologie Biomediche-CNR, Segrate, (MI), Italy

**Keywords:** eNOS, Longevity, Aging, Vascular integrity

## Abstract

Aging is the sum of the deleterious changes that occur as time goes by. It is the main risk factor for the development of cardiovascular disease, and aging of the vasculature is the event that most often impacts on the health of elderly people. The “free-radical theory of aging” was proposed to explain aging as a consequence of the accumulation of reactive oxygen species (ROS). However, recent findings contradict this theory, and it now seems that mechanisms mediating longevity act through induction of oxidative stress. In fact, calorie restriction − a powerful way of delaying aging − increases ROS accumulation due to stimulation of the basal metabolic rate; moreover, reports show that antioxidant therapy is detrimental to healthy aging. We also now know that genetic manipulation of the insulin-like-growth-factor-1/insulin signal (IIS) has a profound impact on the rate of aging and that the IIS is modulated by calorie restriction and physical exercise. The IIS regulates activation of nitric oxide synthase (eNOS), the activity of which is essential to improving lifespan through calorie restriction, as demonstrated by experiments on eNOS knockout mice. Indeed, eNOS has a key role in maintaining vascular integrity during aging by activating vasorelaxation and allowing migration and angiogenesis. In this review, we will overview current literature on these topics and we will try to convince the reader of the importance of vascular integrity and nitric oxide production in determining healthy aging.

## Introduction

Max Rubner’s “rate of living” theory [1] combined mass-specific resting metabolic rate and maximum lifespan of mammalian species to calculate the “lifetime energy potential”: it holds that the pace of life is inversely related to the length of life. Raymond Pearl [2] used this concept to explain longevity variation within species and gave it the “rate of living” label.

The subsequent “free-radical theory of aging” (FRTA) was formulated from the above concepts: Harman [3] postulated that accumulation of free radicals was the prime cause of the sequential alterations characterizing advancing age and the progressive increase in disease and death rates [4].

With the introduction of a “lipids” perspective to the FRTA, it was then asserted that the level of peroxidizability of the cell membrane bilayer was a critical determinant of the severity of cell damage caused by free radicals: in response to attack by free radicals, peroxidation − the susceptibility to which is dictated by the number of single-bonded carbon atoms between the –C=C– units of the fatty acyl chain − generates a strong self-propagating reaction that causes damage to other molecules [5].

The “membrane pacemaker” theory of aging incorporates these concepts to hold that high membrane fluidity and low membrane peroxidizability are the optimal membrane conditions for promoting longevity. This theory arose from the observation that body mass/maximum lifespan in mammals and birds correlates respectively directly and inversely with the levels of C18:1n-9 and C22:6n-3 fatty acids in cellular membranes. This can be explained by the fact that C22:6n-3 is 320-fold more susceptible than C18:1n-9 to peroxidation, while the latter fatty acid has fluidity properties as good as the former [6].

### Lipids, eNOS and exceptional longevity

Exceptional longevity in humans is a complex trait. Long-living individuals have delayed aging and a low incidence of cardiovascular disease [7,8]. We recently reported that in children of nonagenarians the peroxidation index of erythrocyte membrane lipids was significantly lower than in a group of matched controls [9]. It is of particular interest that we found significantly increased levels of palmitoleic acid (C16:1n-7) in the nonagenarians’ offspring, similarly to what was later observed in genetically modified long-living worms [10]. Because these worms were genetically modified in homologue genes of the insulin-like growth factor 1 (IGF1)/forkhead box O3 (FOXO3A) axis, a possible explanation for this finding is that IGF-1 signalling modulates, or is modulated by, the membrane fatty-acid composition [11]. Moreover, it was reported that after chronic thermal or saline stress of yeast, the induced increase in the level of membrane palmitoleic acid was responsible for a reset of heat shock protein (Hsp) release to higher levels [12]. Thus, the high C16:1n-7 detected in the offspring of nonagenarians could be correlated to the low serum level of Hsp70 detected in centenarians’ offspring [13].

In apparent contrast with the FRTA is the finding that the cell membranes of offspring of nonagenarians as well as of long-living DAF2 *Caenorhabditis elegans* mutants have an accumulated amount of endogenous *trans* fatty acids [9] and unpublished data]. Endogenous *trans* fatty acids are an index of endogenous free-radical cellular stress and are produced by endothelial nitric oxide synthase (eNOS)-generated nitrates (NO_2_·), as shown by the lack of *trans*-arachidonic acids in the retinas of eNOS^−/−^ mice [14]. Moreover, calorie restriction has been shown to increase longevity in organisms ranging from yeasts to mammals; it induces the expression of eNOS and mitochondrial biogenesis, which in turn increases oxygen consumption. These effects were abolished in eNOS^−/−^ mice [15]. We therefore speculated that eNOS, nitrate radical stress and the *trans* fatty-acid accumulation observed in nonagenarians’ offspring are all interconnected in the delayed-aging action of calorie restriction, in apparent contrast with the FRTA [16]. The *trans* fatty acids could serve as molecular signals that ultimately induce endogenous defence mechanisms culminating in increased stress resistance and longevity, an adaptive response named hormesis [17].

In agreement with this hypothesis, deletion in worms of mitochondrial proteins such as ISP-1 and NUO-6 induces the oxidative stress necessary and sufficient for promoting longevity: in fact, this effect is abolished by antioxidants and is induced by mild treatment with oxidants [18]. Taken together, these findings question Harman's FRTA and suggest, rather, that reactive oxygen species (ROS) act as essential signalling molecules promoting metabolic health and longevity through an eNOS/nitrate/*trans* fatty acids axis [19]. The degree of oxidative stress could possibly explain this apparent paradox: low stress being protective, whereas massive stress becomes deleterious.

### Calorie restriction, exercise, genetic makeup and eNOS

The beneficial effects of calorie restriction are multiple: it reduces the incidence of tumours and diabetes and the age-related decline in T-lymphocyte proliferation [20]. The effects of calorie restriction can be explained by increased IGF1-insulin signal (IIS) efficiency: in fact, findings on patients with growth hormone receptor deficiency suggest that their high insulin sensitivity could account for the absence of diabetes and very low incidence of cancer seen in these individuals [21].

Furthermore, calorie restriction can be mimicked by genetic manipulation aimed at blocking IIS (i.e., the IGF1/PI3K/AKT/FOXO3A axis): for example, the FIRKO mouse − a carrier of a fat-specific insulin receptor knockout − and *C. elegans* models carrying null mutations of *daf-2* − an IGF1 homologue − and *age-1* − a homologue of the catalytic subunit of mammalian PI3K− all live longer than wild-type animals [22,23]. To be noted, the beneficial effects of *daf-2* and *age-1* null mutations are antagonized by null mutation of *daf-16*, which encodes three members of the FOXO family of transcription factors [23]. Thus, via AKT the IIS is important for controlling eNOS and, hence, human longevity [24].

Genetic variants that are either protective or deleterious for human health can be identified by studying the genetic pool of centenarians: the so called “positive biology approach” [25,26]. Interestingly, apolipoprotein E (APOE) − a variant of which is associated with exceptional longevity in humans across populations − controls the IIS pathway by influencing PI3K [27]. Similarly, the presence of genetic variants of FOXO3A − another member of the IIS − is highly replicable in long-living populations [28-30].

Exercise is inversely correlated with total mortality [31]. An elegant report on athletes undergoing marathon training identified a combination of metabolites (i.e., glycerol, niacinamide, glucose-6-phosphate, pantothenate and succinate) that increased in the plasma in response to exercise; in vitro, these metabolites were able to up-regulate the expression of NUR77, a transcriptional regulator of glucose utilization and lipid metabolism genes [32]. NUR77 is under the control of Ca^2+^/calmodulin-dependent protein kinase (CAMKIV), which is activated by AMPK and has been associated with human exceptional longevity [33,34]. Furthermore, AMPK controls eNOS phosphorylation, which explains the potentiation of eNOS activity by both calorie restriction and physical exercise [24]. AMPK is activated acutely at exercise intensities above ≈ 60% of maximal aerobic capacity [35]. Calorie restriction and exercise both activate mitochondrial biogenesis through activation of AMPK with an eNOS-dependent mechanism, as shown by experiments on eNOS knockout mice [36]. Thus, the beneficial effects on longevity of calorie restriction, genetic makeup and exercise can be explained, at least in part, through eNOS-dependent activation of mitochondrial biogenesis.

### Vascular endothelial dysfunction and eNOS in aging

**Figure 1:**
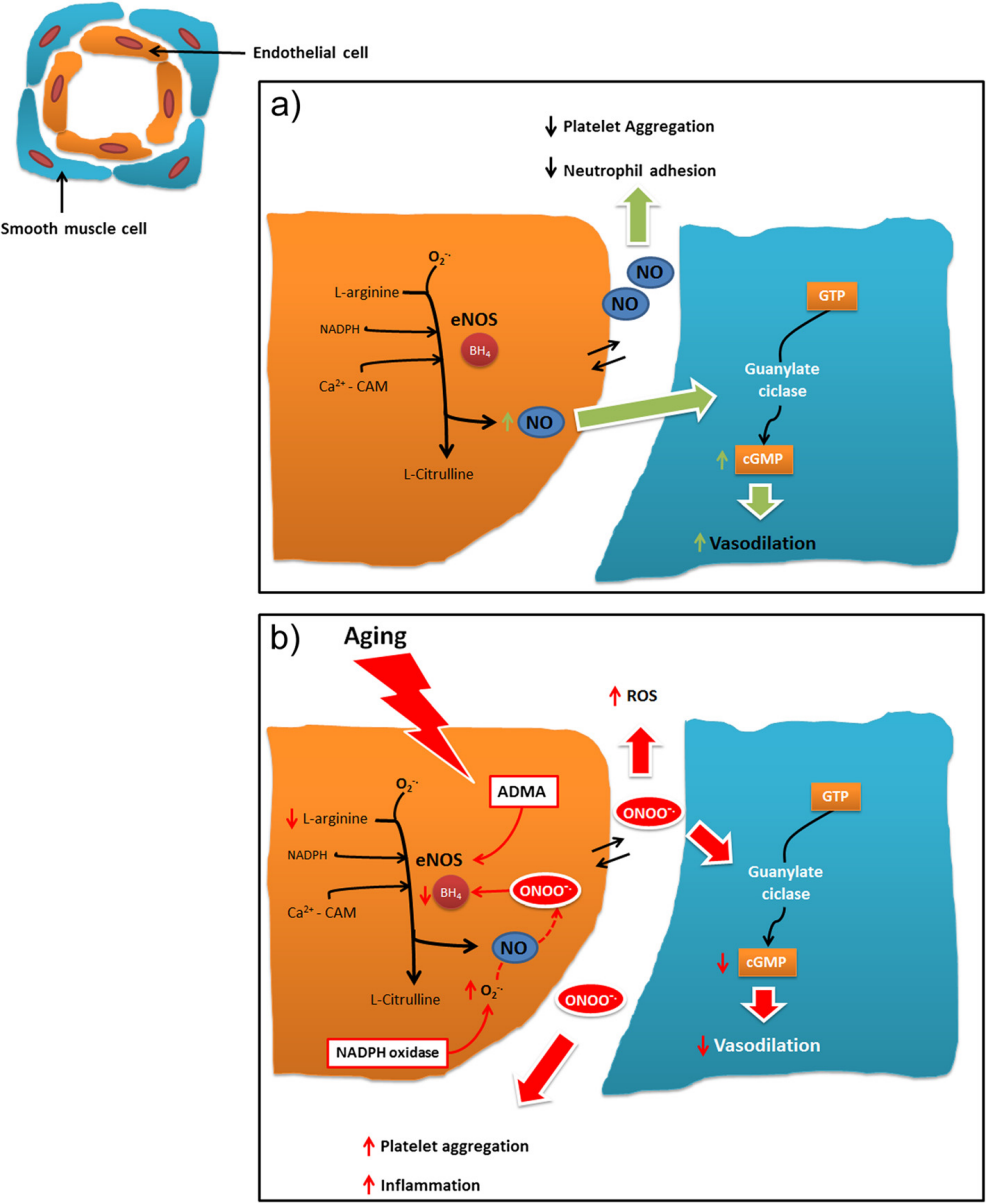
**a) Representative nitric oxide pathway.b) Effects of aging on nitric oxide pathway.** BH_4_ = tetrahydrobiopterin; Ca^2+^ = calcium ion; cGMP = cyclic guanosine monophosphate; eNOS = endothelial nitric oxide synthase; GTP = guanosine triphosphate; NADPH = nicotinamide adenine dinucleotide phosphate; NO = nitric oxide; ONOO^-^ = Peroxynitrite; ADMA = Dimethylarginine; ROS = reactive oxygen species.

Endothelial dysfunction is the hallmark of vascular damage in advancing age [37-41]. Many functions of the vascular endothelium are modulated by NO, which is able to induce smooth muscle relaxation [42-44], the inhibition of platelet aggregation [45], leukocyte adhesion to endothelial cells [46,47] and preservation of endothelial progenitor cell function [48] (Figure 1a). The crucial role of NO in protecting the cardiovascular system during aging was revealed by studies demonstrating that eNOS knockout mice have a premature cardiac-aging phenotype and early mortality [49].

Reduction of NO availability alters vascular homeostasis [50] and is a phenomenon involved in the development of hypertension [50,51], atherosclerosis and thrombosis leading to heart attack and stroke [52-54]. The mechanisms underlying vascular aging are complex and involve multiple pathways [41,55,56] (Figure 1b). During aging, there is a progressive misbalance between NO production − which becomes increasingly reduced − and oxidative stress − which increases without a compensatory enhancement of antioxidant defences [57-59]. As a result, aged vessels have a compromised vasodilatory function, which induces increased vascular resistance and impaired perfusion [60]. Reduced NO production may be dependent upon several mechanisms, including: a) deficiency in eNOS substrates and cofactors, such as l-arginine [61] and tetrahydrobiopterin (BH4) [62]; b) the presence of endogenous eNOS inhibitors, such as asymmetric dimethylarginine (ADMA) [63,64] and analogues of l-arginine present in plasma and various tissues, which have been described as cardiovascular risk factors [65]; and c) lower expression and/or activity of eNOS due to abnormalities in eNOS trafficking to caveolae, to altered eNOS phosphorylation status or to uncoupling of eNOS activity [39,66,67].

An important molecule regulating eNOS activity is sirtuin 1 (Sirt-1), a longevity factor that modulates cellular senescence [68] and promotes endothelium-dependent vasodilation by targeting eNOS for deacetylation [69]. Sirt-1 expression was found lower in endothelial cells from older adults than healthy younger individuals [66,70] in which endothelial vasorelaxation was demonstrated to be positively related to Sirt-1 expression. Calorie restriction − which improves health and slows the aging process − has been reported to induce eNOS expression, improve mitochondrial biogenesis and increase Sirt-1 expression; thus, a positive feedback loop links Sirt-1 and eNOS [71], and activation of SIRT1 may help to reset the activity of eNOS during situations of endothelial dysfunction where NO availability is limited.

BH4 is an essential cofactor for NO synthesis by eNOS. When it is limited − because of a decrease in biosynthesis or an increase in its oxidation − eNOS becomes uncoupled and induces release of superoxide, which, in turn, leads to degradation of NO. Administration of BH4 to older adults caused a selective improvement in endothelial vasorelaxation, demonstrating that BH4 potentially leads to eNOS recoupling in aged human vasculature [72].

Another mechanism that reduces NO bioavailability, hence contributing to vascular endothelial dysfunction with aging, is oxidative stress [73]. Oxidative stress is induced mainly by uncoupling of eNOS, upregulation of the oxidant enzyme nicotinamide adenine dinucleotide phosphate-oxidase (NADPH oxidase) [50,74,75] or increased mitochondrial production of ROS [76-79]. Increased expression of NADPH oxidase has been reported in vessels from aged humans not presenting with other cardiovascular risk factors. The use of NADPH inhibitors protects from age-related endothelial dysfunction [80].

Finally, a reduction in the number of mitochondria and an increase in the generation of dysfunctional proteins have been linked to aging through increased oxidative stress and mitochondrial DNA damage [81]. The mechanisms of mitochondrial oxidative stress in aged endothelial cells include also inhibition of antioxidant defence enzymes, such as manganese superoxide dismutase (MnSOD) [82], decline in reduced glutathione content [83] and dysfunction of the electron transport chain [84]. The mitochondrial enzyme p66shc seems to play an important role in regulation of oxidative stress induction, since mice lacking p66hc present with reduced ROS production, improved endothelial function and increased lifespan [85]. However, conflicting findings exist on the effects of antioxidants on vascular function in elderly humans. For example, a study on vitamin E reported that its administration does not reduce endothelial dysfunction in older adults [86]. In contrast, a more recent clinical trial demonstrated acute reversal of endothelial dysfunction in elderly patients after oral administration of an antioxidant cocktail [87].

## Conclusions

The NO pathway and endothelial dysfunction are part of the principal mechanisms involved in the vascular aging process. A better understanding of the complex interactions between them represents an important target for future research. Therapeutic strategies designed to improve endothelial function or provide an alternative source of NO should be primary aims in the drive to reduce the incidence of cardiovascular disease in the elderly. Moreover, studies need to better investigate the effects of antioxidant therapy on endothelial dysfunction in aging.

## Abbreviations

FRTA: Free-radical theory of aging; IGF1: Insulin-like growth factor 1; FOXO3A: Forkhead box O3; Hsp: Heat shock protein; eNOS: Nitric oxide synthase; ROS: Reactive oxygen species; ISS: IGF1-insulin signal; AMPK: 5' adenosine monophosphate-activated protein kinase; ApoE: Apolipoprotein E; CAMK: Ca^2+^/calmodulin-dependent protein kinase; BH4: Tetrahydrobiopterin; ADMA: Dimethylarginine; Sirt-1: Sirtuin 1; NADPH oxidase: Nicotinamide adenine dinucleotide phosphate-oxidase; MnSOD: Manganese superoxide dismutase.

## Competing interests

The authors declare that they have no competing interests.

## Authors’ contribution

AAP and CV wrote the first draft; subsequent drafts were written by AC, AF, FV, who had the overall supervision of the review processing. All authors read and approved the final manuscript.
